# Empty SV40 capsids increase survival of septic rats by eliciting numerous host signaling networks that participate in a number of systemic functions

**DOI:** 10.18632/oncotarget.27448

**Published:** 2020-02-11

**Authors:** Orly Ben-Nun-Shaul, Rohit Srivastava, Sharona Elgavish, Shashi Gandhi, Yuval Nevo, Hadar Benyamini, Arieh Eden, Ariella Oppenheim

**Affiliations:** ^1^ Department of Hematology, Hadassah Medical Center, Jerusalem, Israel; ^2^ The Hebrew University Faculty of Medicine, Jerusalem, Israel; ^3^ Bioinformatics Unit of the I-CORE Computation Center, The Hebrew University and Hadassah Medical Center, Jerusalem, Israel; ^4^ Department of Anesthesiology, Critical Care and Pain Medicine, Lady Davis Carmel Medical Center, Haifa, Israel

**Keywords:** sepsis, empty SV40 capsids, RNAseq, signaling, cellular functions

## Abstract

Sepsis is an excessive, dysregulated immune response to infection that activates inflammatory and coagulation cascades, which may lead to tissue injury, multiple organ dysfunction syndrome and death. Millions of individuals die annually of sepsis. To date, the only treatment available is antibiotics, drainage of the infection source when possible, and organ support in intensive care units. Numerous previous attempts to develop therapeutic treatments, directed at discreet targets of the sepsis cascade, could not cope with the complex pathophysiology of sepsis and failed. Here we describe a novel treatment, based on empty capsids of SV40 (nanocapsids - NCs). Studies in a severe rat sepsis model showed that pre-treatment by NCs led to a dramatic increase in survival, from zero to 75%. Transcript analyses (RNAseq) demonstrated that the NC treatment is a paradigm shift. The NCs affect multiple facets of biological functions. The affected genes are modified with time, adjusting to the recovery processes. The NCs effect on normal control rats was negligible. The study shows that the NCs are capable of coping with diseases with intricate pathophysiology. Further studies are needed to determine whether when applied after sepsis onset, the NCs still improve outcome.

## INTRODUCTION

Sepsis affects millions of individuals annually worldwide, with a mortality of >25% [1]. It accounts for more than 50% of hospital deaths, with mortality rates of 10–20% for sepsis, 20–40% for severe sepsis, and 40–80% for septic shock [2]. Six to nine million people die annually of sepsis out of close to 30 million cases, while half of the survivors suffer from long-term sequela [3]. One-third die during the following year, and one sixth have severe persistent impairments, including functional limitations (i. e. inability to bathe or dress independently), increase in cognitive impairment and a high prevalence of mental health problems (anxiety, depression and posttraumatic stress disorder). About 40% of patients are re-hospitalized within 90 days of discharge for infection or exacerbation of heart failure [4].

Sepsis occurs when an infection, by bacteria, viruses, fungi or parasites, overwhelms the body defense system. An excessive, dysregulated immune response activates the inflammatory and coagulation cascades causing endothelial damage, which may lead to tissue injury, multiple organ dysfunction syndrome (MODS) and an early demise of the patient [4]. When possible, sepsis is managed by drainage of the infection in conjunction with antibiotic treatment [5]. However, drainage is not always possible, and the rapid development and spread of antibiotic resistant bacteria amplifies the problem. Once sepsis develops, treatment relies on the implementation of supportive care in intensive care units, such as mechanical ventilation, renal replacement therapy and more, in order to prevent or reverse MODS [6].

Sepsis is a great burden on the health system. In the United States alone, 970,000 sepsis patients are admitted to hospitals annually, and the numbers have been rising over the years [2, 7]. In 2013, sepsis accounted for more than $24 billion in hospital expenses, and is currently more than twice those of other conditions and continues to grow at three times the rate of other admissions [2]. Numerous potential drugs, targeted at specific steps of the immunological and coagulation cascades, have been evaluated over decades, with no success [7]. It appears that the complex pathophysiology of sepsis does not respond to single-targeted therapeutic strategies.

**Figure 1:**
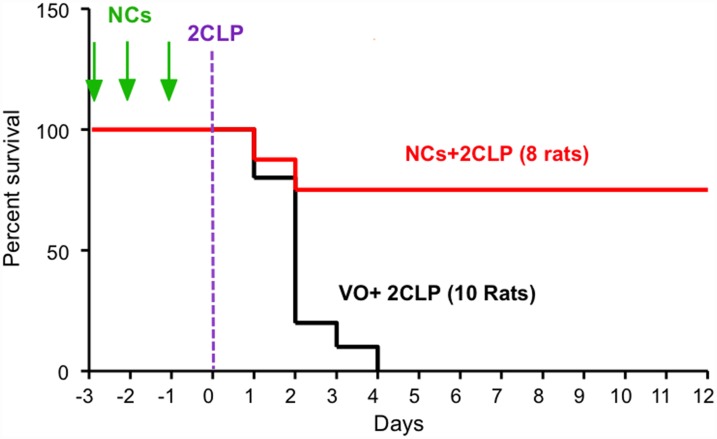
SV40 NCs significantly increase survival of septic rats. Rats were injected with a total dose of 0.3 mg/kg NCs in saline (red) or Vehicle only - saline (black), divided into 3 equal aliquots and injected on 3 consecutive days, (-3,-2,-1), designated by green arrows. All the animals were operated for 2CLP on the 4^th^ day, depicted here as day 0. Statistical analysis using Log-rank (Mantel-Cox) Test for NCs vs. Saline resulted in *p* = 0.0026.

In the present study we tested the hypothesis, based on our earlier unpublished research, that empty SV40 capsids would improve the outcome of sepsis. SV40 is a small, non-enveloped virus of the polyomavirus family. As seen in the crystal structure, the external capsid is a *T*=7 icosahedron, composed of 360 monomers of the viral late protein VP1, arranged in 72 pentamers [8]. The pentamers are formed concurrently with translation of the monomeric subunits by formation of transitory di-sulfide bridges [9]. Twenty years ago we established a procedure for production of empty SV40 capsid, in order to develop a safe gene delivery vector, to be assembled *in vitro* from empty capsids and plasmid DNA of choice [10]. The NCs (also termed Virus Like Particles, VLPs) are very similar in shape to wild-type SV40 under transmission electron microscope - TEM (Figure 3 [10]) and by small angle X-ray scattering - SAXS (Figure 1 [11]). Capsid assembly is a highly cooperative reaction, with a Hill coefficient of ~6 [12]. The NCs are stabilized by calcium ions and disulfide bonds [8, 13].

**Figure 2:**
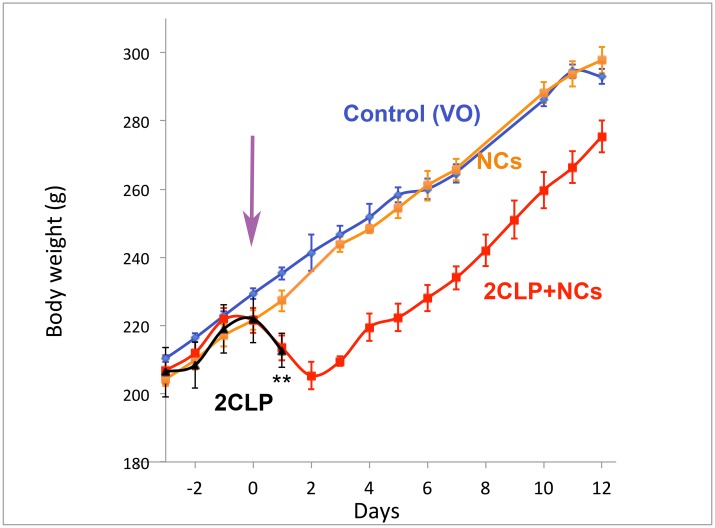
Body weight. Rats were randomly divided into the 4 groups: Control (VO-vehicle only) - blue, Control NCs - orange, 2CLP+VO - black and 2CLP+NCs - red. Injections were performed in 3 equal daily aliquots on days -3,-2,-1, and the 2CLP insult, indicated by the purple arrow, on day 0. Following the insult both 2CLP groups showed rapid decline in weight. Rats of the 2CLP+VO group died within 24–48 hrs, indicated by ^**^, while the 2CLP+NCs rats started re-gaining weight on day 3, and proceeded at the same rate as the controls. Each point indicates an average and standard error of 3–9 rats. The number of rats for each data point is presented in Supplementary Table 1.

**Figure 3:**
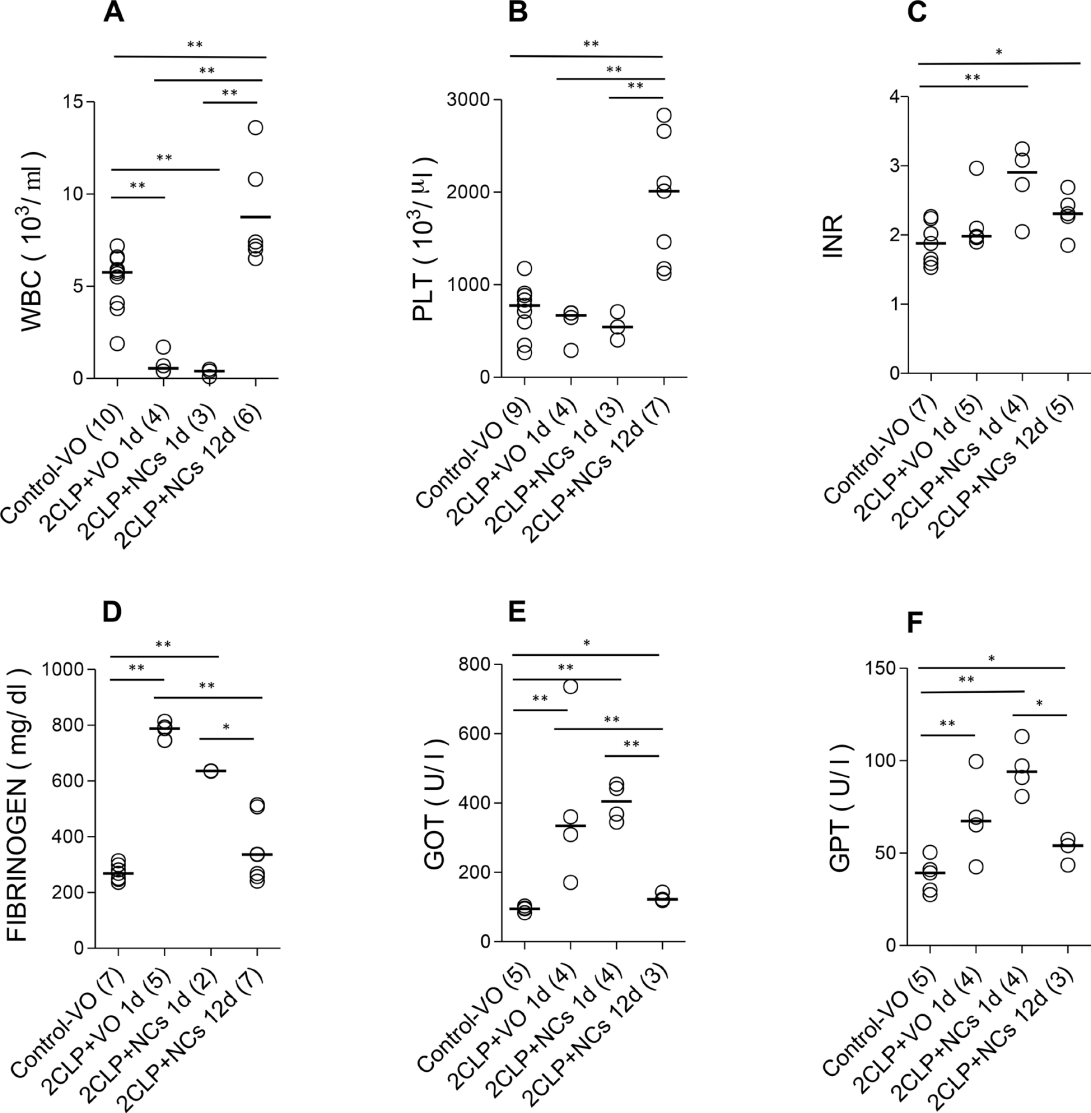
The effect of NCs treatments on routine lab results during disease and recovery progress. Medians of the data collected are indicated. (**A**) White blood cells, (**B**) platelets, (**C**) international normalized ratio, (**D**) fibrinogen, (**E**) glutamic oxaloacetic transaminase, (**F**) glutamic pyruvic transaminase. The number of rats in each group is shown in parentheses. Results of the statistical analyses are presented in Supplementary Table 2. Significant pairwise comparisons are indicated by asterisks, where ^*^ corresponds to 0.05 > *p* > 0.01 and ^**^ to *p* < 0.01.

SV40 recognizes many receptors [14] and has wide tissue and organ tropism. When we investigated the possibility to develop gene therapy for acute kidney injury (AKI) by delivery of Hsp/c70 [15] expressing plasmid to an AKI mouse model, induced by HgCl_2_ [16] or by cisplatin (unpublished), we unexpectedly found that the control empty NCs functioned better than the Hsp70 vector. AKI is characterized by abrupt kidney dysfunction, caused by sepsis, ischemia or nephrotoxic agents. The major underlying mechanisms are apoptosis and necrosis of kidney tubular cells [17]. Pre-treatment by the NCs increased survival from 12 to >60%. Analyses of kidney sections demonstrated arrest of apoptosis, necrosis and renal tubular damage. The underlying mechanism was up-regulation of Hsp/c70 and induction of the PI3K/Akt survival pathway, both seen exclusively in kidney tissue of NCs treated mice [16].

In parallel studies, performed in tissue-cultured cells, we investigated pathways induced by the virus over six hours following infection, before T-antigen expression and the ensued viral propagation. Serine-phosphorylation arrays revealed ~360 of 400 proteins on the array became phosphorylated, implying that the infecting virus triggers an extensive signaling network [18]. Similar signaling was also elicited by the NCs. That study revealed that SV40 and/or its NCs elicit concurrently opposing pathways: cellular stress response, pro-apoptotic host defense, and Akt-1 survival pathway. Remarkably the pathways were robustly balanced, as the infected cells neither apoptosed nor proliferated (in absence of T-antigen) [18].

Here we tested the hypothesis that SV40 NCs will improve outcome in sepsis-induced acute respiratory distress syndrome (ARDS).

## RESULTS

The experiments were performed on peritoneal sepsis in Sprague-Dawley male rats using the widely accepted cecal ligation and double puncture (2CLP) model [19]. In this model the abdomen is opened, the cecum is ligated, then punctured twice before returning it to its position, and the abdomen closed. Severity of the insult is adjustable, depending on location of the ligation and the size of the needle used for punctures [19]. To increase the statistical power of the study we used a severe model (see Materials and Methods), which led to rapid death of the insulted animals (Figure 1).

### The effect of NCs on survival

We first tested whether the NCs affect survival. Rats were randomly divided into 2 groups: both groups underwent the 2CLP operation; one was treated with NCs (in saline) and the other with vehicle (saline) only (VO). The NCs, or the vehicle, were injected through the tail-vein over 3 consecutive days prior to the 2CLP operation. The results (Figure 1), demonstrate a dramatic increase in survival of the NC treated rats. None (0/10) of the 2CLP+VO rats survived beyond 4 days. 8 of the 10 rats of this group died within 48 hours. On the other hand, 6/8 (75%) of the 2CLP rats that received NCs at a total dose of 0.3 mg/kg survived. In spite of the small number of animals, the results were highly significant, *p* = 0.0026, based on Log-rank (Mantel-Cox) Test. Furthermore, these results were consistent with additional experiments, some of which were performed for establishment of the model (not included here) and the others in the extensive disease follow up studies (next section).

### The progress of sepsis and recovery

Rats were randomly divided into 4 groups. Two non-CLP control groups: (I) VO and (II) NC, and two study groups: (III) 2CLP+VO and (IV) 2CLP+NC. As in the previous experiment, the NCs or vehicle were injected through the tail-vein during 3 consecutive days prior to the 2CLP operation.

The clinical condition of the 4 groups of rats was followed over a total period of 12 days. The rats were weighed daily; blood and organs were taken at sacrifice. Because of the time consuming procedures, the study was divided into small groups, including at least one 2CLP+VO and one 2CLP+NC rats. The experiments and data collection were conducted over months, attesting to the reproducibility of the system and results.

Few hours after the operation all the 2CLP-operated rats appeared less active. At 24 hours post operation, their activity further decreased and some were completely inactive. Black circles appeared around the eyes and snout. Additional symptoms were piloerection and soft stools, progressing to diarrhea. Unlike the 2CLP+VO rats, the 2CLP+NC rats began recovering by 48 hours, slowly increasing activity, eating, drinking and gaining weight, which reached a rate similar to that of the control rats by day 3 (Figure 2). NC treatment of control rats did not affect their growth rate. The number of rats represented by each data point is shown in Supplementary Table 1.

Blood data are presented in Figure 3. The data for group II, the non-CLP NC-treated rats (NC), were similar to those of the non-CLP untreated ones (VO). To simplify the presentation, the data for group II are not presented. Data for the 2CLP+VO group are available only for day 1, since most of them were dead on day 2. For the 2CLP+NC group, the data obtained on day 4 were between those of days 1 and 12 days post insult and are not included. Statistical analyses of the data are shown in Supplementary Table 2. Note that results for both groups of 2CLP-insulted animals were consistent with severe sepsis at 24 hours post insult, followed by recovery of the NC-treated survivors.

White blood cell (WBC) count decreased one day after 2CLP in both vehicle and NC-treated groups (2CLP+VO and 2CLP+NC). It increased in the 12-day survivors to significantly higher than normal level, consistent with recovery from the sepsis. Platelet count increased in the 12-day survivors, possibly reflecting enhanced megakaryopoiesis induced by the inflammatory process, as recently described [20]. A significant increase in international normalized ratio (INR) of prothrombin time (evaluating both the extrinsic pathway and common pathway of coagulation) was observed in the 2CLP+NC group one day after the 2CLP insult, but not in the 2CLP+VO group, suggesting a difference in the disease process in the two groups already at that time point. The hematological parameters of the control untreated (VO) animals are consistent with previously reported values for this rat strain [21]. Fibrinogen activation may reflect the acute phase response of both groups of septic rats.

The increase in liver enzymes, glutamic oxaloacetic transaminase (GOT) and glutamic pyruvic transaminase (GPT) indicates liver damage in the sick animals. Results for creatinine and urea were not affected by the 2CLP-operation in either the NC or VO treated groups (not shown).

C-reactive protein (CRP) is an acute phase response protein, markedly elevated during sepsis in humans. However, in the rat, CRP does not participate in the acute phase response [22]. Consistent with that report, the level of CRP did not increase following the 2CLP operation in our study, although our RNAseq experiments showed that acute phase response signaling was the most prominently elevated in both 2CLP-operated groups.

Lactate, a hallmark of inadequate perfusion in human patients, was not affected in the septic rats. The values for lactate of all rats the ranged from 4.1 to 4.8 mM with a standard deviation (StD) of 0.8.

**Figure 4:**
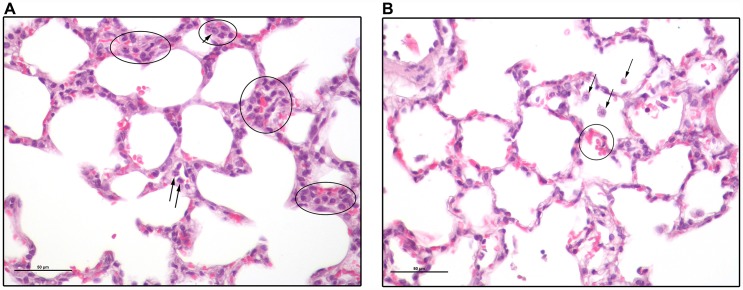
Lung sections of a 2CLP+NCs rat 4 days following the 2CLP-operation. (**A**) Several groups of cells are circled. Most of these are mononuclear and have histiocytic features. They include alveolar macrophages, pneumocytes type II, dendritic cells and monocytes. Some are in alveolar septae, either within capillaries or not, while others are in alveolar spaces. Arrows point to a few cells with lobulated nuclei typical of neutrophils. (**B**) Black arrows point to alveolar macrophages. There are a few RBC in the alveolar spaces indicative of hemorrhage (circled). Bar size is 50 μm.

The lungs are commonly involved in sepsis and are sensitive to injury. Consistent with previous reports [23], our histologic study in the Sprague-Dawley rat presents a picture that is different from typical human ARDS. Lung harvested 24 hours after 2CLP showed no pathologic changes in either 2CLP-operated group. Minor changes were seen on day 4 in lungs of surviving 2CLP+NC rats (Figure 4), including slight increase in the number of alveolar macrophages and a few RBC in alveolar spaces, indicative of mild damage. An increased number of intravascular neutrophils were observed in alveolar capillaries (not shown). No abnormalities were identified in lungs of 2CLP+NC survivors on day 12. Liver histology showed minimal damage in the 2CLP animals (not shown).

### RNAseq studies at 6 hours post-surgery

To obtain an insight into the underlying mechanism of the therapeutic action of NCs on sepsis induced ARDS, we performed RNAseq studies on lung RNA. Mild clinical signs (mostly reduced activity) began to appear at several hours post the 2CLP operation, and were quite severe at 24 hours. By 48 hours most of the vehicle-treated 2CLP rats died. Therefore, RNA for analysis was extracted from rats sacrificed at 6 and 24 hours.

**Figure 5:**
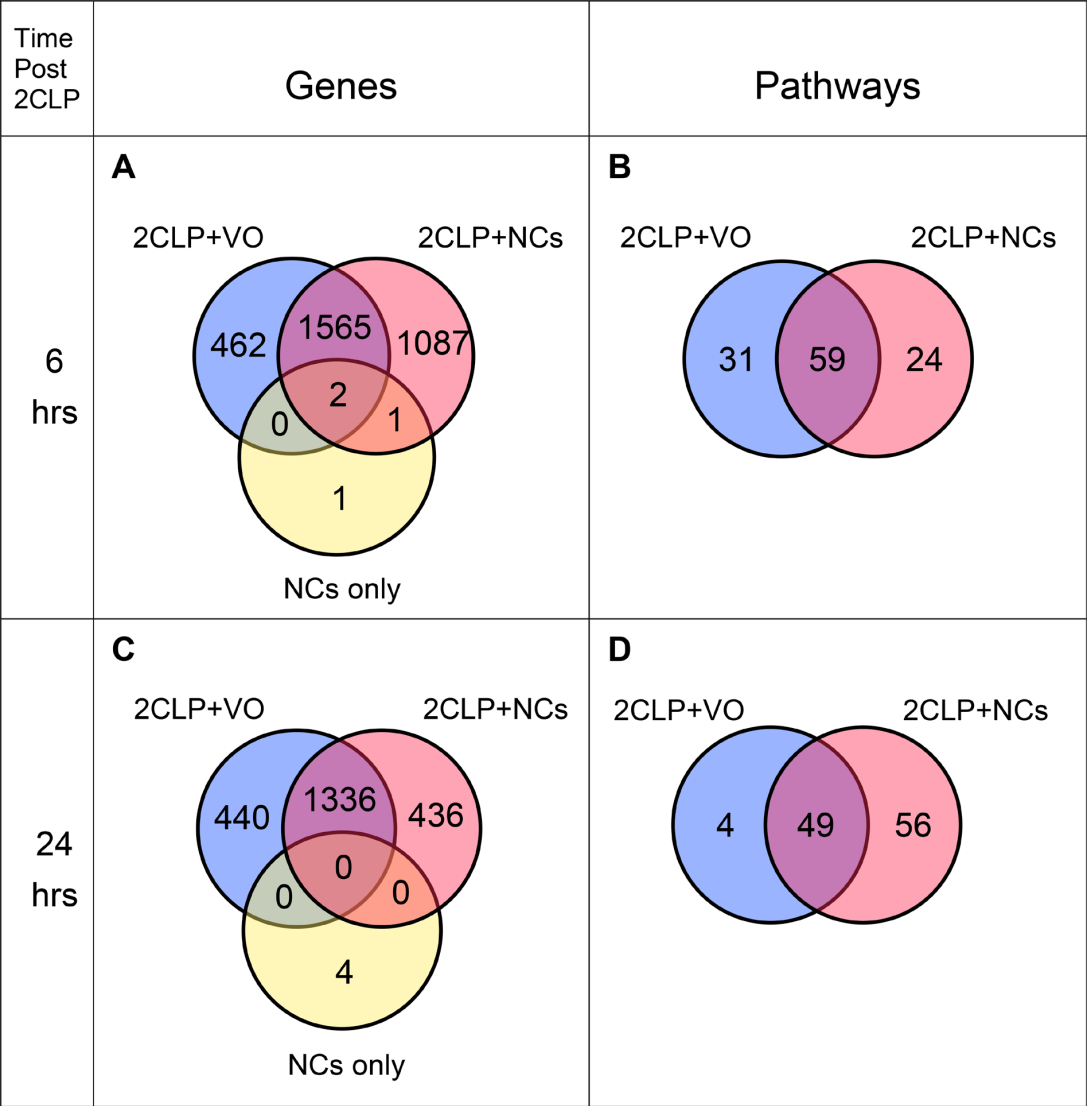
Venn diagrams of expressed genes and enriched pathways. RNAseq was performed on RNA extracted from lungs (3 rats each). (**A**) Genes and (**B**) Enriched pathways for lungs harvested 6 hours post insult. (**C**) Genes and (**D**) Enriched pathways for lungs harvested 24 hours post insult.

Differential expression analysis demonstrated ≥2-fold change, either increasing or decreasing, in thousands of transcripts in septic rats compared to the control (vehicle treated rats). Figure 5A shows that the 2CLP-operation caused, within 6 hours, changes in a total of 2029 transcripts. NC pretreatment reduced the number to 1567, and affected additional 1088 genes, which most likely contributed to the resolution of the disease.

Remarkably, only 4 genes were affected by NC treatment of control rats in comparison to the vehicle treated control (Figure 5A), indicating that perturbation of cellular functions of healthy animals by NCs is minimal. This finding leads us to speculate that administration of NCs to human subjects would cause negligible side effects.

We then performed enriched pathway analyses, using Ingenuity Pathway Analysis (IPA, Qiagen). Figure 5B shows that 59 pathways are common to both 2CLP groups, reflecting their septic state 6 hours post-insult. By that time, NC pretreatment eliminated 31 pathways, which appear only in the 2CLP+VO rats; in addition, the treatment led to the enrichment of 24 pathways unique to the 2CLP+NC group. These 24 pathways (Table 1) represent a range of cellular and systemic functions that most likely participate in the therapeutic effect. We grouped the pathways into four categories: anti-pathogenic and immune responses (pathways #1-10), inflammation and its resolution (pathways #11-14), tissue regeneration (pathways #15-19) and cell proliferation and its regulation (pathways #20-24).

**Table 1:** Ingenuity canonical pathways enriched (BH *p*-value < 0.05) only in the 2CLP+NC group

	Ingenuity canonical pathways	genes	Probability B-H (FDR)	Proposed role in the therapeutic effect of NPs	Refs
1	Toll-like Receptor Signaling	18	8.51E-03	Cytokine induction and activation of iNOS	[24]
2	Super-pathway of Citrulline Metabolism	6	1.95E-02	Anti-pathogen: Nitric oxide production	[25]
3^*^-	iNOS (inducible Nitric Oxide Synthetase) Signaling	11	3.63E-02	Anti-pathogen: Nitric oxide production	[26]
4	CD27 Signaling in Lymphocytes	12	4.47E-02	Central immune regulation	[27]
5	Role of JAK1 and JAK3 in Cytokine Signaling	15	4.27E-02	Cytokine and hormone signaling	[28]
6	Role of JAK2 in Hormone-like Cytokine Signaling	9	4.57E-02	Cytokine and hormone signaling	[28]
7	Role of IL-17A in Psoriasis	6	1.38E-02	Immune function: Activation of defensin	[29]
8	IL-17A Signaling in Airway Cells	18	1.12E-02	Immune function: Activation of defensin	[29]
9^*^-	IL-17A Signaling in Fibroblasts	11	9.55E-03	Immune function: Activation of defensin	[29]
10^*^+	Role of IL-17A in Arthritis	17	7.59E-03	Immune function: Activation of defensin	[29]
11^*^+	RANK (Receptor Activator of Nf-kB) Signaling in Osteoclasts	20	2.88E-02	Nf-kB is a central regulator of inflammation and in the resolution of inflammation	[28]
12^*^+	CD40 Signaling	17	2.29E-02	Signaling for non-canonical NF-kB.	[30]
13^*^+	PPAR (peroxisome proliferator-activated receptor) Signaling	19	2.57E-02	Regulator of inflammatory response	[31]
14	HIF1α (Hypoxia Inducible Factor) Signaling	21	4.79E-02	Metabolic reprogramming in inflammation	[32]
15	Role of NANOG in Mammalian Embryonic Stem Cell Pluripotency	29	5.37E-04	Tissue/organ regeneration: Pluripotency	[33]
16	Mouse Embryonic Stem Cell Pluripotency	22	1.38E-02	Tissue/organ regeneration: Pluripotency	[33]
17	PCP (planar Cell Polarity) pathway	16	9.33E-03	Tissue/organ regeneration: Morphogenesis	[34]
18	GM-CSF Signaling	17	1.38E-02	Bone marrow recovery	[35]
19^*^+	Role of Tissue Factor in Cancer	23	2.88E-02	Tissue recovery: angiogenesis	[36]
20	GADD45 Signaling	8	7.41E-03	Regulation of cell cycle homeostasis	[37]
21	Small Cell Lung Cancer Signaling	18	2.19E-02	Cell proliferation	[38]
22	Pancreatic Adenocarcinoma Signaling	24	1.26E-02	Cell proliferation and metastasis	[38]
23^*^	Ovarian Cancer Signaling	32	6.92E-04	Cell proliferation and metastasis	[38]
24	PTEN (Phosphatase and tensin homolog) Signaling	22	4.79E-02	Suppressing proliferation (Tumor suppressor)	[39]

^*^ Designates pathways that are also enriched at 24 hrs post 2CLP operation.

^*^- Designates that the pathway is predicted to decrease at 24 hours.

^*^+ Designates that the pathway is predicted to increase.

Next, we explored the interactions among gene products that participate in the 24 pathways. The total number of transcripts was 411. As some of those gene products were predicted by the IPA analysis to participate in a number of pathways, the number of distinct gene products was only 164. 140 of these 164 gene products were reported, based on experimental data, to directly interact through signaling regulation. These are presented in Supplementary Figure 1 as nodes. Pairs of nodes are connected by 433 edges, representing direct interactions in signaling pathways. According to IPA definitions their functions are: activation, including many via phosphorylation (*n* = 107), inhibition (*n* = 9), both activation and inhibition (*n* = 8), transcription (*n* = 70), protein-DNA (*n* = 29) without specifying whether the interaction results in activation or inhibition, molecular cleavage (*n* = 6) and ubiquitination (*n* = 1). There are 203 protein-protein interactions without specific designation. Further analysis based on GeneCards, Weizmann Institute of Science (https://www.genecards.org/), revealed that 4 of those are receptor-ligand interaction, 3 are phosphorylation (when one of the interacting proteins is a kinase) and one is inhibition via acetylation. Interactions of these 140 proteins generate a single, high connectivity proteins network (Supplementary Figure 1). Each node in the network represents a gene product, and its size correlates with the number of their interactions (connectivity). The nodes are colored according to their functional categories (see Table 1). Supplementary Figure 1 shows that eight of the proteins participate in all four categories.

### RNAseq at 24 hours post-surgery

Figure 5C shows that at 24 hours post insult the numbers of differentially expressed genes decreased from 2655 to 1772 in lungs harvested from NC treated septic rats (2CLP+NC) and from 2029 to 1776 in untreated septic rats (2CLP+VO). At the same period the number of enriched pathways in 2CLP+VO rats decreased from 90 to 53, with only 4 pathways still unique to this group (Figure 5D). In contrast, in the 2CLP+NC group, enriched pathways increased from 83 to 105. Perhaps more meaningful is the increase in pathways unique to this group, which more than doubled, from 24 to 56, suggesting progression of the recovery process (Figure 5D). Only 8 of the 24 pathways enriched for the NC-treated group at 6 hours (marked by asterix in Table 1) appear at 24 hours, allowing a glimpse into the recovery process.

## DISCUSSION

The present study shows remarkable survival and recovery achieved by pre-treatment with the NCs of 2CLP-insulted rats. Two to three days after the insult, the survivors started gaining weight at a rate similar to the control, non-septic rats. Histological examinations and blood parameters were also consistent with recovery, although some parameters still remained outside the normal limits.

What is the molecular mechanism underlying the therapeutic effect of sepsis by the SV40 NCs? Partial insight may be obtained from the results of the RNAseq analyses at 6 and 24 hours after 2CLP. At 6 hours after the insult 24 enriched pathways were unique to the NCs treated animals. Three of those (Table 1, pathways #1-3) participate in the production of nitric oxide (NO), a powerful antimicrobial agent [25]. In addition, Toll-like Receptors (pathway #1) also induce immune response via cytokine production. Host immune response is also induced by pathways #4-10. CD27 signaling (pathway #4) is critical for T cell expansion, activation and survival [26]. JAK/STAT (pathways #5-6) are central regulators of the immune system and many other functions [38] and pathways #7-10 function in the activation of IL-17, a potent inducer of defensin, a short microbial-killing peptide [27]. Briefly, enriched pathways #1-10 represent the host defense against the infecting pathogens.

Pathways #11-14 participate in inflammation and its resolution. NF-kB (pathways #11-12) has a central role in persistent inflammation, increased risk of cancer [39] and in the resolution of inflammation [40]. The NF-kB pathway found here is the non-canonical one, as seen by participation of Nfkb2 and RelB, as well as the genes coding for the inhibitors of the canonical pathway Nfkbia, Nfkbib and Nfkbiz [41]. We propose that pathways #11-12 function in the resolution of inflammation, since they appear only in the NCs treated septic animals. Pathway #13, PPAR Signaling, activates and represses inflammatory genes [29], while HIF1α (pathway #14) regulates inflammation through metabolic reprogramming [30].

The regeneration and morphogenesis pathways (#15-17) presumably function in the repair of damaged tissues and organs, as part of the recovery process. Likewise, pathway #18, GM-CSF Signaling, leads to bone marrow recovery [33]. Pathway #19, Role of Tissue Factor in Cancer, with its multiple effects on angiogenesis [34], most likely plays a part in the restoration of the damaged endothelium.

Pathways #20-24 function in cell cycle regulation, presumably as part of the organ regeneration process. GADD45 (#20) regulates cell cycle homeostasis [35]. We propose that the tumorigenic pathways #21-23 activate cell proliferation [36], which is balanced in the recovering rats by the potent tumor suppressor PTEN (#24) [37].

Twenty-four hours after the insult the perturbation in gene expression was less prominent (Figure 5C). The reduction in the number of enriched pathways unique to the untreated 2CLP animals from 31 to 4 (Figure 5B, 5D) suggests that their fate has been determined. On the other hand, the number of enriched pathways that are unique to the NC-treated animals more than doubled, from 24 to 56. However, of the initial 24 pathways seen for this group at 6 hours, only 8 are enriched at 24 hours (designated by asterix in Table 1). Seven of 10 anti-pathogenic and immune response pathways (#1-10) disappeared, and 2 additional ones decreased in activity. Only a single pathway of these 10 increased in activity at 24 hours (pathway #10). This finding suggests that the initial “stormy” period has been almost controlled and further ongoing processes that participate in recovery are operating. This explanation is consistent with the interpretation that the non-canonical NF-kB signaling (pathways #11,12) [41], that increases in activity at 24 hours, plays a role in the resolution of inflammation [40] in the rat. Other enriched pathways that present increased activity at 24 hours, in comparison to 6 hours, are those associated with regeneration (pathways #19, 23). We interpret these changes in gene expression as adjustment to the progress of the recovery process, an added virtue of the NC treatment.

The interactions among gene products that participate in the 24 pathways that are exclusive to 2CLP+NC (Supplementary Figure 1) shows a high connectivity network: the 140 distinct genes are linked in 433 interactions. Many of the gene products interact with a number of others. Eight gene products, at the center of the network, participate in all 4 functional categories, and many additional ones are associated with several categories. These findings indicate that the initial signaling events exerted by the NCs are confined to a few regulatory hubs. The protein with highest connectivity (number of associated edges) at the center of the network is the signal transducers and activators of transcription 3 (STAT3), which cascades into diverse signaling pathways. STAT3 may orchestrate its multifaceted effect on signaling via Ca^+2^ [42], a central player in cellular homeostasis [43].

We propose that Ca^2+^ signaling plays a significant central role in the therapeutic process elicited by NCs. This is supported by our earlier study, which indicated that both SV40 and its NCs elicit signaling early following infection through PLCγ [18]. PLCγ is presumably activated by SV40 through its engagement of members of the TAM (Tyro3, Axl and Mer) receptor family [14]. PLCγ is required for oscillatory Ca^2+^ signaling through IP3 receptor (IP3R), a canonical activator of Ca^2+^ release from the ER [44]. In our study [18] inhibition of PLCγ arrested all the other early signals. Additional support is provided by our recent results (unpublished), which demonstrate that the NCs trigger Ca^2+^ signaling soon after adsorption to tissue-cultured cells. Moreover, we found that the NCs attenuate cell death induced by etoposide, an effect that is inhibited by the calcium signaling inhibitor 2-APB.

Interestingly, TAM receptors were shown to play critical roles in inflammation [45], immune homeostasis [46], and other cellular functions [47]. Integrins, which also function as SV40 receptors [14, 48], play a central role in inflammation and its resolution [49].

The harsh model required the NCs to be delivered prior to the insult. Therefore, this model does not accurately represent the clinic set-up. However, it provides an insight into the multiple pathways and functions that the NCs induce, leading to remarkable survival and recovery.

Is pre-treatment an inherent requirement for the NC application? Our preliminary experimental results (not shown) in another disease model, traumatic brain injury (TBI), suggest that this is not the case. That study showed improvement of neurological score of the NC-treated TBI mice, with NCs administration for the first time 3-4 hours after the injury. The dramatic increase in survival in the severe 2CLP model presented here suggests that using a milder model, which mimics more closely human sepsis with 30–50% death, the NCs would alleviate morbidity and reduce mortality when provided after the induction of sepsis. This issue requires further investigation.

A therapeutic protein must be non-toxic to humans. Previous studies strongly suggest that SV40 is safe for human treatment. A great concern was raised in the fifties by the discovery that SV40 contaminated the polio vaccines, together with the finding that T-antigen induced tumors in non-immune animals. The extensive epidemiological studies initiated by NIH (reviewed by Shah [50]) clarified that SV40 is non-pathogenic to immune-competent human individuals [51]. All the more so the NCs, which do not contain the viral genome, do not express T-antigen and cannot propagate.

Another concern in the application of a viral capsid is immune response. Innate immune response might block the therapeutic effect. We found that SV40 evades NK cell cytotoxicity by down-regulating the stress-induced ligand ULBP1 [52]. In addition, acquired immune response may prohibit repeated administration. SV40 was found to be non-immunogenic [53], presumably due to stability of its capsid at acidic pH [13], which enables the virus to reach the ER intact. This is unlike most other viruses that undergo conformational changes in the acidic endosomes [54], which lead to their exposure to lysosomal and proteasomal degradation, followed by antigen display at the host cell surface. Indeed an SV40 viral gene vector was demonstrated to allow repeated administrations to mice, raising high level of antibodies against hepatitis B surface antigen but not against SV40, even after 8 repeated monthly inoculations [55].

The common strategy for drug development is targeting a single (or few) pathways by a specific agent (s). Following many years of failed clinical trials [7], it appears that this strategy is unable to cope with a disease with complex pathophysiology such as sepsis. Our study in the AKI mouse model demonstrated activation of Act and Hsp70 by NC treatment, which account for the prevention of apoptosis and necrosis in kidney tubular cells [16]. In contrast, our present data did not show any change in either signaling protein in the treated septic rats. These findings, and the dynamic adjustment of the therapeutic pathways to the recovery course, lead us to suggest that the effect of the NCs is “tailored” both to the type and to the temporal course of the injury, implying a general homeostatic activity. The homeostatic nature of the NC activity is also manifested in their negligible effect on the normal control rats. We propose that this unique property of the NCs was gained through eons of virus-host coevolution, driven by the ‘selfish’ goal of SV40 to propagate in a healthy organism.

## MATERIALS AND METHODS

### Animals

Male Sprague-Dawley (SD) rats, 7 weeks old (body weight 200+/−10 grams), were obtained from Environ RMS (Israel), Ltd. The rats were housed in a pathogen free (SPF) facility at the Hebrew University Faculty of Medicine. They were allowed to acclimatize for at least 3 days after delivery and were maintained on 12-hour light and dark cycles with ad libitum food and water at all times. Maintenance and research were performed in accordance with the Israeli law for animal experimentation and the National Institutes of Health guidelines for the Care and Use of Laboratory Animals. The research was approved by the Institutional Animal Care and Use Committee (IACUC) of the Hebrew University of Jerusalem, an AAALAC accredited organization, and by ACURO, the Animal Care and Use Review Office of the US Army Medical Research and Material Command.

### 2CLP surgery

Rats were anesthetized with clorketam (75 mg/kg) & cepetor (0.5 mg/kg), administered i. p. (intraperitoneal). The 2CLP operation was performed according to Rittirsch *et al*. [19]. The cecum was ligated above the ileocecal valve to maintain the intestinal continuity. Two perforations were made with a 14G IV catheter (BD Venflon, Becton Dickenson Infusion Therapy, Helsingborg, Sweden).

Rats were resuscitated with normal saline (5ml per 100 g body weight) subcutaneously. Buprenorphine, 0.01–0.05 mg/kg was injected for postoperative analgesia, together with antisedan (Atipemezole hydrochloride 5mg/ml) to discontinue the clorketam and cepetor effect. The rats were returned to their cages immediately after surgery and had access to water and food.

### Production and purification of SV40 NCs

NCs were produced in insect Sf9 cells by baculovirus expressing VP1 [10]. The NCs were harvested at 72 hours post infection [12], before they began assembly around cellular DNA. Nuclear extracts were prepared [56]. DNA was digested with 100 units/ml SAN DNase I (Arcticzymes, https://arcticzymes.com/), which is active at high NaCl concentration, in the presence of 2 mM MgCl_2_.

The NCs (MW=~14.5 mega-daltons) were purified from smaller molecules by extensive stirred-ultrafiltration under Argon, using Biomax (PES) membrane (Millipore, https://www.emdmillipore.com/), with a cut-off at 300 kDa. The concentrated filtrate was repeatedly diluted ~10 fold in 0.5 N NaCl, a total of 7–8 times. At the last ultrafiltration step the purified NCs were concentrated to ~1–3 mg/ml. VP1 purity, estimated by polyacrylamide gel electrophoresis, was >97%. The NCs were stable in 0.5N NaCl at −80° C for over a year.

Before administration to rats, the NC stock was thawed on ice and diluted 3 fold in H_2_O, to 167mM NaCl (normal saline concentration is 154 mM). Dilution was performed just before injection since at this salt concentration NCs disassemble into VP1 pentamers after 20–30 min.

### Animal experimentations

Rats were randomly assigned into one of 4 groups: I. vehicle only (VO) - saline control group, II. NC only control group, III. 2CLP+VO and IV. 2CLP+NCs. 2CLP was performed on day 0. NCs (or VO) were administered via the tail vein at a total dose of 0.3 mg/kg, in 3 equal aliquots (0.1 mg/kg daily) for 3 consecutive days, prior to the 2CLP operation (days -3,-2,-1). Tail vein injections, either NCs or saline, were at a total volume of 200 μl. Follow-up began on day 0, no later than 6 hours after operation. From day 1 on the rats were observed daily, at the same hour, and studies (weight and sacrifice, as relevant) were conducted according to a weekly plan. Because the 2CLP procedure is time consuming, no more than 4 rats were operated on the same day, including at least one 2CLP+VO and one 2CLP+NCs. At the indicated time-points rats were sacrificed following anesthesia with clorketam (75 mg/kg). Blood was withdrawn from the heart with heparin or EDTA citrate, for the different tests.

### Clinical evaluation

Complete blood count (CBC) and blood biochemistry were performed at the biochemistry laboratories of Hadassah Medical Center (calibrated for human parameters). Liver enzymes, GOT and GPT, were measured using Reflotron strips (Roche, https://www.roche.com), following the manufacturer’s protocol. Left lungs were fixed in 10% formaldehyde, paraffin embedded, and slides were prepared for histological examination. Right lungs were frozen in liquid nitrogen and stored at −80° C.

### Statistical analyses of the clinical data

The number of animals in each group was very small, 3–4, except for the untreated rats (VO group), which included up to 10. We therefore chose a nonparametric test, Kruskal–Wallis (https://www.statsdirect.com/help/Default.htm#nonparametric_methods/kruskal_wallis.htm) rather than ANOVA. The Kruskal–Wallis test, like all nonparametric tests, uses ranks rather than raw data, and does not require that the variables be normally distributed. The analysis shows (Supplementary Table 2) that the treatments of the various experimental groups had a significant effect on each of the six parameters tested; four of the comparisons were highly significant (*p* < 0.01). All six were followed by *post-hoc* pairwise comparisons according to Conover [57].

### Histological studies

Histological studies were performed by Ori Brenner, B. V. Sc., University of Pretoria, Onderstepoort, School of Veterinary Medicine, Diplomate ACVP. Lung slides, stained with hematoxylin and eosin (H&E), were analyzed using a Nikon E400 microscope, a Nikon DS-FiS camera and NIS elements software. The slides were examined under conventional magnifications (×2, ×4, ×10, ×20, ×40).

### RNA preparation and library formation and sequencing

RNA was purified from three frozen lungs for each of the four groups (VO, NC, 2CLP+VO, 2CLP+NC), a total of 12 extracts for each time point, using RNEasy Kit (Qiagen, https://www.qiagen.com/us). Lysates from lungs were prepared according to manufacturer’s protocol. An RNA aliquot of each lung was further purified on RNeasy column, evaluated for quality in TapeStation, using RNA ScreenTape kit (Agilent Technologies, (https://www.agilent.com), and quantified in Qubit apparatus (Qubit^®^ DNA HS Assay kit, Invitrogen).

Libraries were prepared from the RNA samples by SENSE mRNA-Seq Library Prep Kit (https://www.lexogen.com). The 12 libraries were barcoded and pooled for multiplex sequencing (1.5 pM total including 1.5% PhiX control library). The pooled DNA was loaded on NextSeq 500 High Output v2 kit (75 cycles) cartridge (https://www.illumina.com) and sequenced on Illumina NextSeq 500 System, using sequencing conditions of 75 cycles, single-read.

Library preparation and sequencing were performed at the Core Facility of the Hebrew University Faculty of Medicine.

### Bioinformatics analyses

#### Differential expression analysis

Raw reads were processed according to the library protocol recommendations, including trimming off their first 9 bases, using the FASTX package (version 0.0.14, http://hannonlab.cshl.edu/fastx_toolkit/), trimming low quality bases and adapter sequences from reads ends with cutadapt (version 1.12, https://cutadapt.readthedocs.org/en/stable/), then trimming off the last 6 bases and filtering out low quality reads, using a quality threshold of 20 over 90 percent of the read’s positions. Only reads of length 15 and above were kept.

The processed fastq files were mapped to the rat transcriptome and genome using TopHat (v2.0.14, http://ccb.jhu.edu/software/tophat/index.shtml). The genome version was Rnor6, with annotations from Ensembl release 84. Since many reads mapped close to known genes, but outside the gene boundaries, mapping data was used to extend the boundaries of known genes, using the Cufflinks package (version 2.2.1, http://cole-trapnell-lab.github.io/cufflinks/). Quantification was done using htseq-count (version 0.6.0, https://htseq.readthedocs.io/), given the final genes annotations file (GTF) with extended gene boundaries. Normalization and differential expression were done with the DESeq2 package (version 1.12.4) [58]. Genes with a sum of counts less than 10 over all samples were filtered out prior to normalization, then size factors and dispersion were calculated. Differential expression was calculated using default parameters. Significance threshold was taken as false discovery rate (FDR, B-H *p*Value) of less than 0.05 [59]. Transcripts were further filtered to include only those increased or decreased at least 2-fold (2-fold change, FC2).

#### Enriched pathways analysis

Enriched pathways analysis was performed using Ingenuity Pathway Analysis (IPA) (QIAGEN Inc., https://digitalinsights.qiagen.com/products-overview/discovery-insights-portfolio/content-exploration-and-databases/qiagen-ipa/).

Pathways enriched in each of the two 2CLP-insulted groups, 2CLP+VO and 2CLP+NP, were first compared with the common background of control VO rats. Next, significantly enriched pathways (probability B-H (FDR) *p* Value < 0.05) of the two groups, 2CLP+VO vs VO and 2CLP+NP vs VO, were compared with one another. Enriched pathways were filtered for those with FDR B-H *p*-Value < 0.05 [59].

#### Gene interaction network

The network of interactions among gene products was generated using Cytoscape [60]. Nodes represent gene products and edges represent interaction between gene products. Interaction data was extracted from IPA as follows: the 164 unique genes that participate in the 24 IPA canonical pathways, enriched only in 2CLP+NC, were connected using only direct and experimentally observed interactions, which included the following relationship types: activation, inhibition, activation and inhibition, transcription, protein-DNA (without designation whether the interaction led to activation or repression), molecular cleavage, ubiquitination and protein-protein interactions. 140 out of the 164 genes have generated a network with 541 edges. Filtering for redundancy and self-interactions resulted in 433 unique pairs of gene products that interact in at least one interaction type. One interaction type was assigned to each of the 433 gene pairs, resulting in a network of 140 nodes and 433 undirected edges.

RNAseq studies were performed at the Bioinfomatics unit of the Hebrew University and Hadassah Medical Center.

#### Availability of data and materials

The RNAseq data obtained in this study have been deposited in NCBI’s Gene Expression Omnibus [61] and are accessible through GEO Series accession number GSE125277 (https://www.ncbi.nlm.nih.gov/geo/query/acc.cgi?acc=GSE125277). The data obtained from lungs harvested 6 and 24 hours post 2CLP insult are under accession numbers GSE109041 and GSE125276 respectively.

Additional data obtained IPA, such as upstream regulators, are available upon request from A. O.

## SUPPLEMENTARY MATERIALS



## Abbreviations

2-APB: 2-aminoethoxydiphenyl borate
AKI: acute kidney injury
Akt: protein kinase B
ARDS: acute respiratory distress syndrome
Ca^+2^: Calcium
2CLP: cecal ligation and 2 punctures
CRP: C-reactive protein
ER: endoplasmic reticulum
GADD45: growth arrest and DNA damage
GOT: glutamic oxaloacetic transaminase
GPT: glutamic pyruvic transaminase
HIF1α: Hypoxia-inducible factor 1-alpha
Hsp/c70: heat shock/constitutive protein
INR: international normalized ratio
i.p.: intra-peritoneal
IP3R: inositol trisphosphate receptor
IPA: Ingenuity Pathway Analysis
MODS: multiple organ dysfunction syndrome
NC: empty SV40 nano-capsid
NF-kB: nuclear factor kappa-light-chain-enhancer of activated B cells
NO: nitric oxide
NK: natural killer
PI3K: phosphoinositide 3-kinase
PLCγ: phosphoinositide phospholipase C gamma
PTEN: phosphatase and tensin homolog
RBC: red blood cells
RNAseq: RNA sequencing
SAXS: small angle X-ray scattering
STAT3: signal transducers and activators of transcription 3
StD: standard deviation
SV40: simian virus 40
*T*: triangulation number
T-antigen: tumor antigen
TAM: Tyro3, Axl and Mer
TBI: traumatic brain injury
TEM: transmission electron microscope
ULBP1: UL16 binding protein 1
VLP: virus-like particles
VO: vehicle only
VP1: viral protein 1
WBC: white blood cells
