# Supplementary material for: Empty SV40 capsids increase survival of septic rats by eliciting numerous host signaling networks that participate in a number of systemic functions
Source: Oncotarget. 2020 Feb 11;11(6):574–88. doi: 10.18632/oncotarget.27448 (PMC7021236; doi:10.18632/oncotarget.27448)
Supplement: Supplementary file 1 [file oncotarget-11-574-s001.pdf]

# Empty SV40 capsids increase survival of septic rats by eliciting numerous host signaling networks that participate in a number of systemic functions

## SUPPLEMENTARY MATERIALS

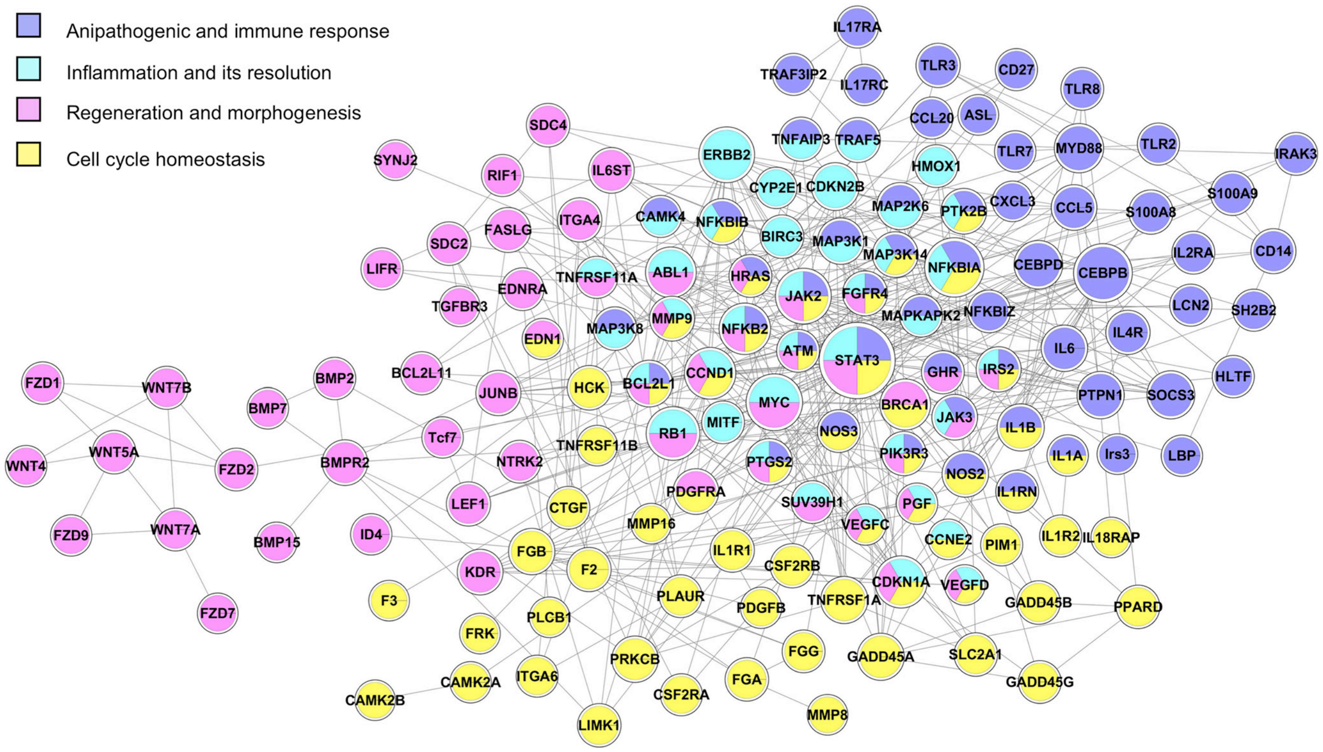


**Supplementary Figure 1: Network of interactions among gene products that participate in the 24 pathways enriched exclusively in the 2CLP+NCs group 6 hours after the 2CLP insult.** The nodes represent gene products and their size correlates with their connectivity (number of their interactions). They are color-coded according to the functional categories that appear in Table 1. Data was extracted from IPA and the network generated using Cytoscape.

**Supplementary Table 1: Body weight-number of rats weighed at each time point**

| Day | -3 | -2 | -1 | 0 | 1 | 2 | 3 | 4 | 5 | 6 | 7 | 8 | 9 | 10 | 11 | 12 |
|---|---|---|---|---|---|---|---|---|---|---|---|---|---|---|---|---|
| Control-VO | 7 | 7 | 7 | 7 | 7 | 3 | 7 | 7 | 4 | 4 | 4 | 0 | 0 | 4 | 4 | 4 |
| Control-VLPs | 7 | 7 | 7 | 7 | 7 | 7 | 7 | 7 | 7 | 7 | 7 | 7 | 7 | 7 | 7 | 7 |
| 2CLP+VO | 3 | 3 | 3 | 3 | 3 | dead | | | | | | | | | | |
| 2CLP+VLP | 9 | 9 | 12 | 12 | 12 | 6 | 4 | 9 | 3 | 3 | 3 | 3 | 3 | 3 | 3 | 3 |

**Supplementary Table 2: Statistical analyses of the effect of treatments on blood parameters**

| Blood parameter / *Parameter *p*-value / Groups compared | WBC | Platelets | INR | Fibrinogen | GOT | GPT |
|---|---|---|---|---|---|---|
| | ***0.0004*** | ***0.0025*** | ***0.0356*** | ***0.0022*** | ***0.0051*** | ***0.0105*** |
| Untreated+VO vs 2CLP+VO 1d | ***0.0003*** | 0.1553 | 0.2226 | ***p < 0.0001*** | ***p < 0.0001*** | **0.0024** |
| Untreated+VO vs 2CLP+VLPs 1d | ***0.0001*** | 0.1801 | ***0.0035*** | ***0.0034*** | ***p < 0.0001*** | ***p < 0.0001*** |
| Untreated+VO vs 2CLP+VLPs 12d | ***0.0004*** | ***0.0004*** | **0.0363** | 0.1257 | **0.0194** | **0.049** |
| 2CLP+VO vs 2CLP+VLPs 1d | 0.4687 | 0.9602 | 0.0545 | 0.2465 | 0.3169 | 0.0882 |
| 2CLP+VO vs 2CLP+VLPs 12d | ***p < 0.0001*** | ***0.0001*** | 0.3642 | ***0.0001*** | **0.0099** | 0.2288 |
| 2CLP+VLPs 1d vs 2CLP+VLPs 12d | ***p < 0.0001*** | ***0.0002*** | 0.2517 | **0.0326** | **0.0017** | **0.0114** |

*The numbers in *Italics* present *p* values obtained by the Kruskal–Wallis test (https://www.statsdirect.com/help/Default.htm#nonparametric_methods/kruskal_wallis.htm). The pair-wise comparisons between groups within each blood parameter were performed according to Conover [1]. VO denotes vehicle only. *p*-values are colored according to their level of significance: ***Italics* bold underlined** – highly significant ($p < 0.01$); **Bold** underlined – significant ($0.05 > p > 0.01$); regular – not significant ($p > 0.05$).

## REFERENCES


1. Conover WJ. Practical Nonparametric Statistics. Hoboken, New Jersey: Wiley; 1999.
